# Editorial: The Role of Reactive Oxygen Species in Chemical and Biochemical Processes

**DOI:** 10.3389/fchem.2021.642523

**Published:** 2021-02-19

**Authors:** Giuseppe Vitiello, Loredana Serpe, Alfonso Blázquez-Castro

**Affiliations:** ^1^ Department of Chemical, Materials and Production Engineering, University of Naples Federico II, Naples, Italy; ^2^ CSGI – Center for Colloids and Surface Science, Florence, Italy; ^3^ Department of Drug Science and Technology, University of Turin, Turin, Italy; ^4^ Department of Biology, Autonomous University of Madrid, Madrid, Spain

**Keywords:** chemical process, nanomaterial, redox activity, ROS, reactive oxygen species

Reactive oxygen species (ROS) are small reactive molecules that play critical roles in the regulation of various cell functions (Forman et al., 2010), chemical (Lam et al., 2020) and biological (Belovolova, 2020) processes. ROS are relatively short-lived molecules that contain oxygen atoms and display half-lives (t_1/2_) in the range of nanoseconds to hours. In many chemical and biological processes, they are usually formed as molecules, ions, or radicals, such as hydrogen peroxide (H_2_O_2_), singlet oxygen (^1^O_2_), hydroxyl radical (^•^OH), hydroperoxyl radical (HOO^•^), superoxide radical (^•^O_2_^−^), hypochlorite ion (ClO^−^), or peroxynitrite (ONOO^−^) (Lushchak, 2014). Depending on the specific type, ROS can play significantly different roles, participating in many important processes (Schieber and Chandel, 2014) and reacting with various target molecules. In biological and biochemical processes, ROS are natural products of oxygen metabolism, playing a key role in cell signal transduction and homeostasis. Under environmental pressure, a dramatic increase in ROS levels can cause oxidative stress (Das and Roychoudhury, 2014), leading to cellular damage or triggering various diseases, including neurological disorders (Balance et al., 2019), cardiovascular diseases (Chen et al., 2019), or different kinds of inflammation (Silwal et al., 2020; Terrone et al., 2020) and cancer (Weinberg et al., 2019). Furthermore, thanks to the high reactivity, ROS are involved in many (photo)chemical processes, which are the basis of several technological applications (Tapeinos and Pandit. 2016; Andina et al., 2017; Nosaka and Nosaka, 2017; Yang et al., 2019).

This Research Topic “The Role of Reactive Oxygen Species in Chemical and Biochemical Processes” collects five articles on the latest advances in understanding the role of ROS in many processes.

Sikora et al. propose a review on the application of redox boronate-based molecular probes, as emerging effective tools for the detection and quantitation of peroxynitrite and hydroperoxides (Sikora et al.). Thanks to their promising advantages, in terms of reaction kinetics, stoichiometry, and mechanisms of action, with respect to the widely used probes based on reduced fluorescent dyes, the boronate probes represent a meaningful opportunity for studying *in vivo* the role of biological oxidants in pathological conditions, including cancer, neurodegeneration, and cardiovascular diseases. This review focuses on the recent progress in the design of boronate-based probes, describing their reactivity and offering many examples of their real use in chemical, enzymatic, and biological systems.

Undoubtedly, cardiovascular health and disease is a major research and clinical area. ROS have been implicated in the genesis and progression of cardiovascular disease, due to their reactive nature. However, in the past years, more and more physiological signaling roles have been adjudicated to ROS in the regulation of cardiovascular function. Alhayaza et al. contribute with a review detailing the relationship between ROS and endothelial cells, master regulators of the cardiovascular system (Alhayaza et al.). In this review, the complex and intricate mechanisms linking endothelial cell metabolism and energy sources (carbohydrates and lipids) with ROS fine-tuned production to promote different physiological outcomes (e.g., angiogenesis or vasodilation) are clearly structured and presented. Finally, some hints as to the connection between ROS production and epigenetics in endothelial cells are provided to conclude the review.

New, out-of-the-box approaches to generate ROS in a controlled way in different biological systems are in development to complement the classical photodynamic systems, in use since more than one century ago. In their mini-review, Carrasco et al. introduce the fundamentals of a particularly appealing approach: plasmonic excitation of metal nanoparticles (Carrasco et al.). Making use of the collective photoexcitation of conduction band electrons in the nanoparticle, it is possible to promote a subpopulation of those electrons to high-energy states. These so-called hot electrons can engage in ROS production, which, in turn, can be employed to modulate biological function through cellular redox targets. The fundamentals, advantages, and some tentative redox biological applications of these plasmonic hot electrons are introduced in this mini-review.

Liang et al. propose an original research article on the development of a novel light-activated tool for targeted generation of singlet oxygen to induce cell death, exploiting a genetically encoded fluorogen-activating protein opportunely complexed with a unique dye molecule that acts as a powerful photosensitizer upon interaction with the protein (Liang et al.). The proposed strategy leads to a localized production of ^1^O_2_ in distinct subcellular regions at consistent per-cell concentrations, determining a light dose-dependent cytotoxic response and inducing apoptotic or necrotic cell death as a function of subcellular location, including the nucleus, the cytosol, the endoplasmic reticulum, the mitochondria, and the lipid membrane. This study offers a new insight into the role of ^1^O_2_-generating photosensitizing processes in inducing targeted cell death and contributes to access the spotlight on the tolerance and survival mechanisms in studies of oxidative stress in clonal cell populations.

The original research article by Mazuryk et al. shows relevant results to understand the role of organic and inorganic components of airborne particulate matter (PM) in provoking adverse health effects (Mazuryk et al.). The *in vitro* biological effects of organic and inorganic PM fractions were investigated on A549 lung epithelial cells by using a novel and interesting experimental protocol. A significant influence of a PM inorganic part on oxidative stress generation was observed. Specifically, oxidative stress was induced by the PM organic fraction with small and moderate PM concentrations for a shorter incubation time (24 h) and by the PM inorganic fraction with increasing PM concentrations for a prolonged incubation time (up to 18 days, mimicking the chronic exposition), resulting in a significantly different profile of ROS generation.

In conclusion, it has been a pleasure to edit this exciting topic of Frontiers in Chemistry. The issue brings together a variety of articles on the key role of these highly reactive molecules, helping to increase the understanding on their involvement in many natural or induced bio-(photo)chemical processes, which are also fundamental for many applications in the biomedical field. The editors hope that the articles will be of interest for researchers in the field of biological and physical chemistry. The articles will contribute to improving the knowledge and understanding of the fundamental properties of ROS.

## References

[B1] Andina D. Leroux J. C. Luciani P. (2017). Ratiometric fluorescent probes for the detection of reactive oxygen species. Chem.-A Eur. J. 23, 13549–13573. 10.1002/chem.201702458 28561437

[B2] Balance W. C. Qin E. C. Chung H. J. Gillette M. U. Kong H. (2019). Reactive oxygen species-responsive drug delivery systems for the treatment of neurodegenerative diseases. Biomaterials 217, 119292. 10.1016/j.biomaterials.2019.119292 31279098 PMC7081518

[B3] Belovolova L. V. (2020). Reactive oxygen species in aqueous media. Optic Spectrosc. 128, 932–951. 10.1134/S0030400X20070036

[B4] Chen Q. Wang Q. Zhu J. Xiao Q. Zhang L. (2019). Reactive oxygen species: key regulators in vascular health and diseases. Br. J. Pharmacol. 176, 1279–1292. 10.1111/bph.13828 PMC5867026 28430357

[B5] Das K. Roychoudhury A. (2014). Reactive oxygen species (ROS) and response of antioxidants as ROS-scavengers during environmental stress in plants. Front. Environ. Sci. 2, 53. 10.3389/fenvs.2014.00053

[B6] Forman H. J. Maiorino M. Ursini F. (2010). Signaling functions of reactive oxygen species. Biochemistry 49 (5), 835–842. 10.1021/bi9020378 20050630 PMC4226395

[B7] Lam P. L. Wong R. S. M. Lam K. H. Hung L. K. Wong M. M. Yung L. H. (2020). The role of reactive oxygen species in the biological activity of antimicrobial agents: an updated mini review. Chem. Biol. Interact. 320, 109023. 10.1016/j.cbi.2020.109023 32097615

[B8] Lushchak V. I. (2014). Free radicals, reactive oxygen species, oxidative stress and its classification. Chem. Biol. Interact. 224, 164–175. 10.1016/j.cbi.2014.10.016 25452175

[B9] Nosaka Y. Nosaka A. (2017). Generation and detection of reactive oxygen species in photocatalysis. Chem. Rev. 117, 11302–11336. 10.1021/acs.chemrev.7b00161 28777548

[B10] Schieber M. Chandel N. S. (2014). ROS function in redox signaling and oxidative stress. Curr. Biol. 24, R453–R462. 10.1016/j.cub.2014.03.034 24845678 PMC4055301

[B11] Silwal P. Kim J. K. Kim Y. J. Jo E. K. (2020). Mitochondrial reactive oxygen species: double-edged weapon in host defense and pathological inflammation during infection. Front. Immunol. 11, 1649. 10.3389/fimmu.2020.01649 32922385 PMC7457135

[B12] Tapeinos C. Pandit A. (2016). Physical, chemical and biological structures based on ROS‐sensitive moieties that are able to respond to oxidative microenvironments. Adv. Mater. 28, 5553–5585. 10.1002/adma.201505376 27184711

[B13] Terrone G. Balosso S. Pauletti A. Ravizza T. Vezzani A. (2020). Inflammation and reactive oxygen species as disease modifiers in epilepsy. Neuropharmacology 167, 107742. 10.1016/j.neuropharm.2019.107742 31421074

[B14] Weinberg F. Ramnath N. Nagrath D. (2019). Reactive oxygen species in the tumor microenvironment: an overview. Cancers 11 (8), 1191. 10.3390/cancers11081191 PMC6721577 31426364

[B15] Yang B. Chen Y. Shi J. (2019). Reactive oxygen species (ROS)-based nanomedicine. Chem. Rev. 119, 4881–5498. 10.1021/acs.chemrev.8b00626 30973011

